# Supplementary material for: Older adults detect happy facial expressions less rapidly
Source: R Soc Open Sci. 2020 Mar 25;7(3):191715. doi: 10.1098/rsos.191715 (PMC7137944; doi:10.1098/rsos.191715)
Supplement: Supplementary Table 1 [file rsos191715supp1.doc]

Supplementary Table 1. Mean (with standard error) proportions of correct responses in each target condition among young and older adults.

|  | Young | Older |
| --- | --- | --- |
| Normal anger  Normal happiness  Anti-anger  Anti-happiness | 0.81(0.03)  0.79(0.03)  0.68(0.03)  0.72(0.03) | 0.79(0.04)  0.76(0.03)  0.70(0.03)  0.69(0.03) |
